# Supplementary material for: Timing matters: modeling the effects of gestational cannabis exposure on social behavior and microglia in the developing amygdala
Source: Neuropsychopharmacology. 2025 Jul 23;50(11):1655–64. doi: 10.1038/s41386-025-02173-5 (PMC12436615; doi:10.1038/s41386-025-02173-5)
Supplement: Supplementary file 1 — Supplemental Methods and Supplemental Table 1 [file 41386_2025_2173_MOESM1_ESM.pdf]

Supplemental Methods:

<u>Immunohistochemistry</u>

For fluorescent imaging, slide-mounted sections were washed in phosphate buffer saline (PBS) and blocked with either 10% bovine serum albumin (BSA) in PBS + 0.4% Triton X-100 (PBS-T) for 1 h. Slides were incubated in primary antibody solution (5% BSA in PBS-T) overnight. The following day, slides were incubated in secondary antibody solution (5% BSA in PBS-T) for 2 h and coverslipped with ProLong Diamond Antifade (Thermo Fisher Scientific).

For 3,3′-diaminobenzidine (DAB) staining, slide-mounted sections were washed in Tris-buffered saline (TBS; 0.05M, pH 7.6), incubated in 0.3% hydrogen peroxide in TBS for 30 min at room temperature. Sections were blocked with 10% BSA in TBS + 0.4% Triton X-100 (TBS-T). Sections were incubated in primary antibody in solution (5% BSA in TBS-T) overnight. The following day, sections were incubated in biotinylated secondary antibody for 1 h, followed by incubation in ABC reagent (1:500 dilution; Vectastain Elite ABC Kit, Vector Laboratories) in TBS-T for 1 h, and visualized using DAB chromagen or nickel-enhanced DAB chromagen (0.05% 3,3′-diaminobenzidine, 0.2% nickel (II) sulfate, 0.006% hydrogen peroxide; all from Sigma-Aldrich). The DAB reaction was allowed to proceed until completion, as confirmed under a microscope. Sections were counterstained with either hematoxylin or methyl green, dehydrated in an ascending ethanol series, defatted in xylene, and coverslipped with DPX mounting medium.

Primary antibodies used included the following: Rabbit anti-Iba1 (1:1000; Wako Cat#019-19741, RRID:AB_839504), mouse anti-bromodeoxyuridine (BrdU) (1:500; BD Biosciences Cat#347580, RRID:AB_400326), Secondary antibodies used included the following: biotinylated anti-rabbit (1:500; Vector Laboratories Cat#BA-1000, RRID:AB_2313606), biotinylated anti-mouse (1:500; Vector Laboratories Cat#BA-2000, RRID:AB_2313581), Alexa Fluor 488- or 594-conjugated antibodies to rabbit (488 Cat#A21206, RRID:AB_2535792; 594 Cat#A21207, RRID:AB_141637), or mouse (488 Cat#A21202, RRID:AB_141607; 594 Cat#A21203, RRID:AB_2535789).

<u>Unbiased stereological cell counting</u>

Every third section (45μm thick) was used for analysis for a total of four sections, and both hemispheres of the amygdala were quantified. The boundaries of each region were drawn with a 4x objective, referencing a neonatal rat atlas [42], and the optical fractionator method was used to quantify microglia and BrdU+ cells at 40x magnification, using a 100μm x 100μm counting grid with a 250μm x 250μm sampling grid for microglia and a 50μm x 50μm counting grid with a 250μm x 250μm sampling grid for BrdU+ cells. The optical dissector height was set to 12μm with a 2μm guard zone on the top and bottom for both quantifications. Microglia were counted based on the presence of an observable cell body within the designated counting region and determined to be phagocytic if the microglia contained an observable phagocytic cup that was distinctly identifiable from the cell body. BrdU+ cells were counted if the nuclear staining was uniformly dark and was within the designated counting region.

<u>Quantification of cell density</u>

The surface tool was used to create volumetric boundary of either the developing amygdala for neonatal studies, or the medial amygdala in adults. To determine the density of microglia within these regions, individual microglia were counted using the automatic spots function which counted microglia based on their Iba1 positive soma and cell size of approximately 13μm. Density was then calculated by dividing the number of microglia by the volumetric area of the determined region. Proliferating microglial density was determined by using the spots function to automatically count cells that were double positive for both Iba1 and BrdU, and then dividing that total by the area of the volumetric boundary.

<u>Developmental battery</u>

Weight: Weights of rats were taken daily from P0-90.

Dam Latency to Retrieve Pups: On P5 each pup was placed one at a time opposite the nest and the time it took the dam to retrieve the pup was measured. Data was rank ordered within litter with the latency being assigned a value 1-12, with the rank then being divided by the total litter size to scale the data with the fastest being 1/12 and the slowest being 12/12.

Surface Righting: From P5-10 each pup was placed on its back on a flat surface. The time for the pup to return to its four limbs was recorded with a cutoff time of 30 seconds.

Developmental Locomotion: From P5-15 each pup was placed on a flat surface in the center of an underlaid circle 13cm in diameter. The time for all four legs of the pup to exit the circle was recorded with a cutoff time of 30 seconds.

Negative Geotaxis: From P5-15 each pup was placed face down on a ramp with a 25-degree incline that was covered with a wire mesh to enable traction. The time for the pup to turn 180 degrees with its head and trunk oriented to the top of the incline was recorded with a cutoff time of 30 seconds.

Wire Hang: From P10-15 each pup's forepaws were placed against a horizontal wire rod suspended above a padded bin. The duration of time each pup hung from the wire was recorded with a cutoff time of 10 seconds.

Juvenile behavioral battery

The behavioral battery was performed as previously described [27, 43]. All behavior testing took place during the dark phase of the animal's light/dark cycle under red light illumination. Rats were weaned on P21 and housed in same-sex, same-treatment sibling pairs. All videos were recorded

Open Field: On P26 rats were placed in an open field (78 × 78 cm, 40 cm high; polycarbonate walls), underlaid with a grid delineating perimeter and center regions. The rats behavior was video recorded for 10 min and center time and gridline crosses were scored manually offline.

Novel Object Recognition: On P27 rats were placed in the same arena used for open field testing and were exposed to a pair of identical objects placed on opposite ends of the arena for 5 min. After this exposure, rats were placed back in their home cages for 1 h and then were returned to the arena and exposed to a now familiar object as well as a novel object. Rat behavior was video recorded during both sessions and manually scored offline using a virtual stopwatch for the time spent investigating each object, and the recognition index was calculated (time with novel object / time with novel object + time with familiar object). The position of objects in the arena and the order of object exposure was counterbalanced between treatment groups.

Social Recognition: On P29 rats were singly housed in a test cage identical to their home cage with ad libitum access to food and water for 2 h to allow for habituation. A sex and age matched stimulus rat was then placed into the cage with the test rat for 4 min and the number of time the experimental animal spent interacting with the stimulus animal was recorded. The stimulus rat was then removed, and the test rat remained in the test cage for a retention interval of 20 min. The original familiar stimulus rat and a novel stimulus rat were then placed into the test cage with the test rat. Rat behavior was video recorded during both sessions and manually scored offline for the time the test rat spent investigating each of the stimulus rats and recognition index was calculated (time with novel rat / time with novel rat + time with familiar rat).

Social Play: On P30 rats were isolated for two hours and then same-sex, same-treatment non-sibling pairs of rats were placed in an enclosure (49 × 37 cm, 24 cm high) with TEK-Fresh cellulose bedding. One animal is head marked with a permanent marker to delineate the two. Rats were allowed to acclimate for 2 min, then video recorded for 10 min. Videos were then scored offline,

with the animal performing a given behavior receiving a score for that behavior. The behaviors are as follows:

- Pouncing: Scored when one animal launches itself toward the other's nape or back
- Pinning: Scored when a pouncing animal flips the other completely onto its back
- Boxing: Scored when both rats rear up on their hind legs and paw at each other

Total play, a summation of all these play events, was calculated for each animal.

Elevated Plus Maze: On P32 rats were individually placed on an elevated plus-shaped polycarbonate apparatus with two open (102.5 × 12 cm) and two enclosed arms (102.5 × 12 cm, 45.5 cm high) elevated 72cm from the ground. Rats were recorded using a video camera and analyzed using ANY-maze video tracking software (Stoelting, Wood Dale, IL, USA) for 5min. The time spent by each animal in the open arms compared to the closed arms was then used as a measure of anxiety-like behavior.

Quantification and statistical analysis

To assess possible litter effects for all studies that were performed during gestational timepoints, data was initially analyzed *a priori* using the dam as the n (i.e. response of all pups in a litter averaged). No differences were found between significant data whether the dam was the n or whether individual offspring were the n, indicating no significant litter effects in our experiments. Data including multiple timepoints were analyze *a priori* using a three-way analysis of variance (ANOVA) to determine if there was a main effect of sex, and when no main effect of sex was found, sex was collapsed, and data were then analyzed using a two-way ANOVA for age and treatment. Data including multiple experimental groups were analyzed using two-way ANOVA for treatment and sex when appropriate. A one sample t-test against a theoretical value of 0.5 to determine if recognition differed from random chance was used.

Supplemental Methods:

Supplemental Table 1: Summary table of behavioral effects of THC treatment paradigms

Summary table showing the behavioral assays performed, the age they were performed at, and the effects either postnatal or prenatal THC had on them for both the developmental battery as well as the battery performed during the juvenile period.

Behavior	Age	Postnatal THC (P0-7)	Prenatal THC (GD15-21)
**Developmental Battery**			
**Body Weight**	P0-90	No Effect	No Effect
**Dam Latency to Retrieve Pups**	P5	**Decreased latency to retrieve females**<br>**Increased latency to retrieve males** *	No Effect
**Surface Righting**	P5-10	No Effect	No Effect
**Developmental Locomotion**	P5-15	No Effect	No Effect
**Negative Geotaxis**	P10-15	No Effect	No Effect
**Wire Hang**	P10-15	No Effect	No Effect
**Behavioral Battery**			
**Open Field; Center Time**	P26	No Effect	No Effect
**Open Field; Line Crosses**	P26	No Effect	No Effect
**Novel Object Recognition**	P27	No Effect	No Effect
**Social Recognition**	P29	**Decreased social recognition in females**<br>**Decreased social recognition in males** *	**Decreased social recognition in females**<br>**Decreased social recognition in male** *
**Social Play**	P30	**Increased social play in females**<br>**Increased social play in males** *	**No effect in females**<br>**Decreased social play in males** *
**Elevated Plus Maze**	P32	No Effect	No Effect
